# Polyhydroxyalkanoate-Based Microparticles for Enhanced Photostability and Controlled Release of Pyraclostrobin

**DOI:** 10.3390/polym18111380

**Published:** 2026-06-02

**Authors:** Mi-Jin Kim, Hansol Kim, Min Chul Park, Seong-Bo Kim, Dong-Jin Jang, Sung Tae Kim

**Affiliations:** 1 Department of Nanoscience and Engineering, Inje University, Gimhae 50834, Republic of Korea; kmj0110020@naver.com (M.-J.K.); hsk@inje.ac.kr (H.K.); 2 Department of Innovative Pharmaceutical Sciences and Engineering, Inje University, Gimhae 50834, Republic of Korea; 3 Department of Pharmacy, Inje University, Gimhae 50834, Republic of Korea; mcpark@inje.ac.kr; 4 Bio-Living Engineering, Global Leaders College, Yonsei University, Seoul 03722, Republic of Korea; seongbo.kim@yonsei.ac.kr; 5 Department of Bio-Health Technology, College of Biomedical Science, Kangwon National University, Chuncheon 24341, Republic of Korea

**Keywords:** polyhydroxyalkanoate, pyraclostrobin, microparticles, photostability, drug release

## Abstract

Photodegradation under ultraviolet irradiation significantly limits the efficacy of agrochemicals, leading to reduced field performance and increased environmental burden. Biodegradable polymer-based delivery systems have emerged as a promising strategy to address these limitations. Polyhydroxyalkanoate (PHA)-based microparticles (MPs) encapsulating pyraclostrobin (PYR), a model fungicide, were prepared using an emulsion–solvent evaporation method. The formulations were characterized by physicochemical properties, encapsulation efficiency, and release kinetics. Photostability under UV irradiation and antifungal activity against *Aspergillus oryzae* were systematically evaluated. As results, the MPs exhibited uniform size distribution and high encapsulation efficiency. Physicochemical analyses confirmed the physical incorporation of PYR within the PHA matrix without chemical alteration. The PHA matrix suppressed UV-induced photodegradation, enhancing photostability. In addition, the system demonstrated sustained release without an initial burst, with release rates dependent on polymer composition. This controlled release behavior resulted in prolonged antifungal activity. Based on these results, PHA-based microencapsulation provides an effective and sustainable strategy to enhance the stability and efficacy of photolabile agrochemicals, offering a versatile platform for advanced pesticide delivery.

## 1. Introduction

Pesticides, encompassing fungicides, herbicides, and insecticides, are indispensable to modern agriculture, safeguarding crop productivity against a broad spectrum of pathogens and pests [1]. However, the practical performance of many active ingredients is compromised by their limited environmental stability, particularly under ultraviolet (UV) irradiation in field conditions. Such susceptibility to photodegradation results in rapid chemical breakdown, leading to diminished biological activity and shortened persistence after application [2]. Consequently, repeated applications are often required to maintain efficacy, exacerbating both environmental burden and economic cost [3,4]. To mitigate these limitations, substantial efforts have been directed towards the development of advanced formulation strategies [5,6,7]. Among these, polymer-based microencapsulation has emerged as a versatile platform capable of enhancing the physicochemical stability of active ingredients while enabling controlled release profiles, as demonstrated for compounds such as lambda-cyhalothrin [8,9,10]. Despite these advances, the materials conventionally employed in encapsulation systems frequently present inherent limitations, including poor biodegradability, risk of environmental accumulation, and manufacturing complexity. Moreover, their capacity to effectively attenuate UV-induced degradation remains insufficient, thereby constraining their utility in field applications [11,12,13,14,15].

In this context, polyhydroxyalkanoates (PHAs) have garnered increasing attention as a class of microbially derived, biodegradable polyesters with considerable potential for sustainable delivery systems [16,17]. Their intrinsic hydrophobicity and tunable physicochemical properties render them particularly well-suited for the encapsulation of poorly water-soluble compounds [18]. Beyond their role as passive carriers, the dense polymeric matrix of PHAs may also function as a physical barrier that modulates the interaction between encapsulated actives and external stressors, including UV irradiation. Consistent with this premise, our preliminary studies demonstrated the efficient encapsulation of therapeutic agents within PHA-based systems [19,20]. These observations suggest that extending PHA-based encapsulation to agrochemical applications may offer a viable strategy to enhance the environmental stability of pesticides. Nevertheless, the exploitation of PHAs for pesticide stabilization remains largely unexplored.

Herein, we report the development of a PHA-based microencapsulation system for pyraclostrobin (PYR), a widely used strobilurin fungicide known for its pronounced photostability. The resulting formulations were systematically evaluated in terms of physicochemical characteristics, encapsulation efficiency, and release behavior. In addition, their resistance to UV-induced degradation was quantitatively assessed to elucidate the protective capacity of the PHA matrix.

## 2. Materials and Methods

### 2.1. Materials

PHA was purchased from Helian Polymers BV (Belfeld, The Netherlands). PYR and benzophenone (internal standard) were both purchased from Tokyo Chemical Industry (TCI, Tokyo, Japan). Polyvinyl alcohol (PVA), used as a surfactant, was purchased from Duksan General Science (Ansan, Republic of Korea). Chloroform, used as the oil phase in the emulsion, was purchased from Daejung Chemicals and Metals (Siheung, Republic of Korea). *Aspergillus oryzae* was obtained from the Korean Collection for Type Cultures (KCTC, Jeongeup, Republic of Korea) and cultured on potato dextrose agar (PDA, BD Difco™, Franklin Lakes, NJ, USA) for antifungal activity assays. All other reagents and chemicals used were of analytical grade.

### 2.2. Preparation of Standard Solutions and Quantitative Analysis

A stock solution of PYR (10 mg/mL) was prepared in chloroform. Working standard solutions were prepared by diluting the stock solution with acetonitrile to concentrations of 1.56–200.00 µg/mL. A benzophenone stock solution (10 mg/mL), used as an internal standard, was prepared in dimethyl sulfoxide (DMSO, Duksan General Science, Ansan, Republic of Korea) and further diluted to a final concentration of 50 µg/mL.

All samples were quantitatively analyzed using high-performance liquid chromatography (HPLC, Agilent 1100 series, Agilent Technologies, Santa Clara, CA, USA). HPLC analysis was performed using a C_18_ column (Supersil^®^ 120 ODS II, 4.6 × 150 mm, 5 µm; LB Science, Seoul, Republic of Korea). The mobile phase consisted of acetonitrile and water (60:40, *v*/*v*) at a flow rate of 1 mL/min. The injection volume was 10 µL, and detection was carried out at 275 nm with a UV detector. All data were processed using Agilent ChemStation software (Rev. B.04.03, Agilent Technologies).

### 2.3. Method Validation

Precision and accuracy were evaluated by analyzing samples at different concentrations using intra- and inter-day validation methods. For intra-day analysis, eight different concentrations were measured five times within a single day. For inter-day analysis, the same concentrations were also analyzed on five consecutive days. Precision was expressed as the relative standard deviation (RSD), and accuracy was determined by comparing the measured concentrations with the nominal values. The limit of quantification (LOQ) was defined as the lowest concentration with a signal-to-noise ratio of at least 10, precision below 20%, and accuracy within the range of 80–120% [21].

### 2.4. Microencapsulation of PYR Using PHA

Microencapsulation of PYR were prepared using an oil-in-water (*o*/*w*) single emulsion solvent evaporation technique [22]. Briefly, 30 mg/mL PHA solution in 2 mL of chloroform was mixed with PYR solution in 1 mL of chloroform at various amounts to form the organic phase. The organic phase was then added to 30 mL of 5% (*w*/*v*) PVA solution and emulsified using a homogenizer (HG-15D, DAIHAN Scientific, Seoul, Republic of Korea) at 15,000 rpm for 2 min. The resulting emulsion was stirred at 500 rpm for 3 h under a fume hood to allow evaporation of the organic solvent. The microparticles were collected by centrifugation at 5000 rpm for 30 min at 4 °C and washed with double-distilled water to remove residual PVA. The obtained particles were stored at 4 °C until further use.

The encapsulation efficiency (EE) of PYR microparticles (PYR-MPs) was quantitatively calculated as the percentage of PYR encapsulated within the microparticles relative to the initial amount of PYR used. All experiments were performed in triplicate (*n* = 3).

### 2.5. Physicochemical Properties of PYR-MPs

#### 2.5.1. Size Measurement of PYR-MPs

The particle size and size distribution of PYR-MPs were measured using a particle size analyzer (PSA, Anton Paar PSA 990, Anton Paar, Graz, Austria). Each particle size was expressed as the mean diameter, and the size distribution was represented by the SPAN value. All measurements were performed in triplicate (*n* = 3), and the results are presented as mean ± standard deviation [23].

#### 2.5.2. Scanning Electron Microscopic Analysis of the PYR-MPs

Each morphology of PYR-MPs was examined using a scanning electron microscope (SEM, AIS2300C, SERON Technologies, Uiwang, Republic of Korea). All samples were dried in a vacuum oven and sputter-coated with platinum at 15 mA for 150 s prior to imaging, which were obtained at an accelerating voltage of 20.0 kV.

#### 2.5.3. Fourier-Transform Infrared Spectroscopic Measurement of PYR-MPs

PYR, blank MPs (without PYR), and PYR-MPs were analyzed using Fourier-transform infrared spectroscopy (FT-IR, Varian 640-IR FT-IR spectrometer, Agilent Technologies, Santa Clara, CA, USA), respectively. The samples were mixed with potassium bromide (KBr) at a sample-to-KBr ratio of 1:30 and compressed into pellets prior to analysis. The spectra were recorded over the range of 4000–400 cm^−1^ with a resolution of 4 cm^−1^ with 64 scans.

#### 2.5.4. Differential Scanning Calorimetric Measurement of PYR-MPs

PYR, blank MPs, and PYR-MPs were analyzed using a differential scanning calorimeter (DSC, DSC-60, Shimadzu, Kyoto, Japan). Approximately 5–7 mg of each sample was placed in a standard aluminum pan and sealed prior to analysis. An empty aluminum pan was used as a reference. The samples were heated from 30 °C to 200 °C at a heating rate of 10 °C/min under an air atmosphere at a flow rate of 40 mL/min. Two heating cycles were performed for each sample.

#### 2.5.5. X-Ray Diffraction Measurement of PYR-MPs

X-ray diffraction (XRD) analysis was performed for PYR, blank MPs, and PYR-MPs using an XRD system (Ultima IV, Rigaku, Tokyo, Japan) with Cu-Kα radiation at 40 kV. The diffraction patterns were recorded over a 2*θ* range of 2–70° at a scanning rate of 1°/min [24].

### 2.6. Storage Stability of PYR-MPs

Colloidal storage stability of MPs with different PYR ratios was evaluated at three temperature conditions (4 °C, 25 °C, and 54 °C) for 2 weeks [25]. Both particle size and size distribution were measured using a PSA, as described above. All measurements were performed in triplicate (*n* = 3), and the results are presented as mean ± standard deviation.

### 2.7. Photostability of PYR Encapsulated in MPs

The photodegradation resistance of PYR in MPs was evaluated under UV exposure in a given period of time [26]. Free PYR solution was used as the control, and suspensions of PYR-MPs with different ratios were prepared at final PYR concentrations of 50, 100, and 150 µg/mL and dispersed in glass Petri dishes. Each sample was placed at distances of 20 and 65 cm from a 36 W UV lamp and exposed to UV irradiation at room temperature. For the 20 cm condition, samples were collected at 0, 2, 4, 6, 8, and 10 min, whereas for the 65 cm condition, samples were collected at 0, 2, 4, 6, 8, 10, 20, and 30 min. The remaining PYR concentration was quantified using HPLC. The half-life (DT_50_) of PYR under UV irradiation was determined based on a first-order kinetic model [27].

### 2.8. In Vitro Release of PYR from MPs

In vitro release studies were performed using a dialysis membrane method [28]. Briefly, PYR-MPs at different ratios were prepared to contain the same amount of PYR (4 mg). The samples were then loaded into Slide-A-Lyzer™ G3 dialysis cassette with a molecular weight cut-off (MWCO) of 10,000 Da (Thermo Fisher Scientific, Rockford, IL, USA). Each cassette was immersed in 50 mL of release medium. The release medium consisted of phosphate-buffered saline (PBS, pH 7.4) with ethanol (70:30, *v*/*v*). The experiment was conducted at 25 °C using a shaking incubator (JSSI-100T, JSR Research Inc., Gongju, Republic of Korea) at 100 rpm. At predetermined time intervals (0, 3, 5, 7, 9, 12, 24, 36 and 60 h), 1 mL of the release medium was withdrawn and replaced with an equal volume of the same medium. Each amount of released PYR was quantified at various time points using HPLC system. The cumulative release was expressed as the percentage of PYR released relative to the initial amount of PYR in the MPs [29]. Additionally, the release data were analyzed using zero-order, first-order, Higuchi, and Ritger–Peppas kinetic models [30].

### 2.9. Antifungal Activity Assays of PYR

The antifungal activity of PYR released from MPs was evaluated using a disk diffusion assay [31,32]. Prior to antifungal activity assay, *Aspergillus oryzae* was cultured on PDA plates at 25 °C for 3–5 days. The fungal spores were then harvested in PBS and adjusted to a concentration of 1 × 10^6^ spores/mL. Then, 100 μL of the spore suspension was uniformly spread onto PDA plates. Sterile paper disks (6 mm in diameter) were loaded with 10 μL of the release samples harvested at 12 h and 60 h and placed on the plate surface. The release medium (PBS 70% and ethanol 30%, *v*/*v*) was used as a control. The plates were incubated at 25 °C, and the diameter of the inhibition zone was measured.

### 2.10. Statistical Analysis

Statistical analysis between two groups was performed using Student’s *t*-test with the Social Science Statistics (https://www.socscistatistics.com/tests/studentttest/calculator/, accessed on 5 March 2026). For comparisons involving three or more groups, one-way analysis of variance (ANOVA) was conducted using the StatsKingdom (https://www.statskingdom.com/180Anova1way.html, accessed on 6 March 2026). Statistical significance was established at *p*  ≤  0.05.

## 3. Results

### 3.1. Validation of HPLC Method

**Figure 1:**
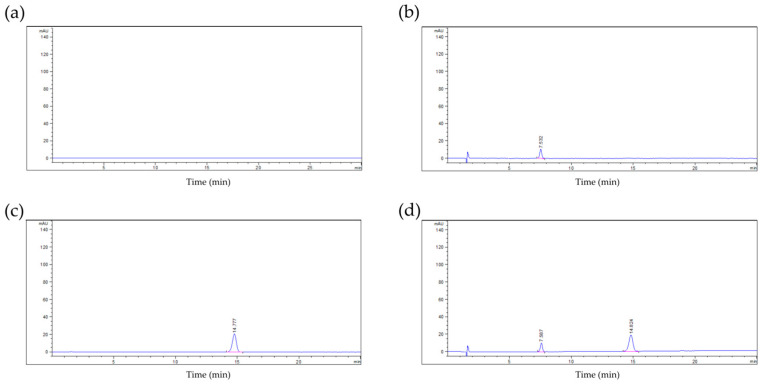
Representative chromatograms of (**a**) a blank, (**b**) benzophenone (the internal standard), (**c**) PYR, and (**d**) a benzophenone–PYR mixture.

Under chromatographic conditions, the retention times of benzophenone, an internal standard and PYR were 7.5 and 14.8 min, respectively (Figure 1). The calibration curve exhibited the linearity over the concentration range of 1.56–200.00 μg/mL, described by the regression equation as follows: y = 0.3006(±0.0037)x + 0.1182(±0.0790) with a correlation coefficient (R^2^) of 0.9998 (±0.0001). Both intra- and inter-day precision values were below 10%, indicating high reproducibility of the method. The accuracy ranged from 86.00 to 101.84% and 81.06–103.70% for intra- and inter-day measurement, respectively (Table 1), confirming the reliability of the assay across the tested concentration range. The LOQ was determined at 1.56 μg/mL, yielding a signal-to-noise (S/N) ratio greater than 10 with precision below 20%, and accuracy within 80–120% [33]. Collectively, these results demonstrate that the developed HPLC method is reliable and suitable for the quantitative analysis of PYR as a model fungicide.

### 3.2. Size and Distribution of PYR-MPs

**Figure 2:**
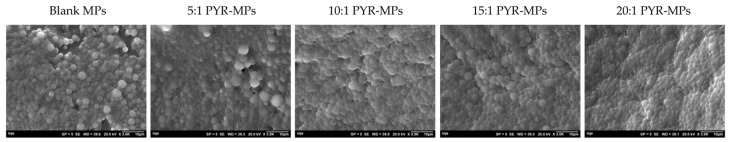
Scanning electron microscopy images of the PYR-MPs prepared at various PHA:PYR ratios (scale bar = 10 μm).

PYR-MPs were prepared using an oil-in-water (O/W) solvent evaporation method, and their physicochemical properties were evaluated (Table 2). The mean particle size of blank MPs was 2.40 ± 0.09 μm. The average particle size was consistently maintained at approximately 2.41 μm regardless of the PHA-to-PYR ratio (5:1–20:1), indicating that drug loading had a negligible effect on the size. Each SPAN values, indicating particle size distribution, was also low across all formulations, suggesting a narrow and uniform size distribution. This observation was also supported by scanning electron microscopy (SEM) images (Figure 2), which confirmed the formation of MPs with uniform globular morphology and size distribution. Meanwhile, the encapsulation efficiency (EE) of PYR in MPs ranged from 74.13% to 75.45% across all formulation ratios, demonstrating minimal variation with respect to polymer-to-drug ration. These results indicate that the emulsion-based fabrication method enables stable particle formation and efficient drug encapsulation with consistently high EE (approximately 74%).

### 3.3. Fourier-Transform Infrared Spectroscopic Analysis

**Figure 3:**
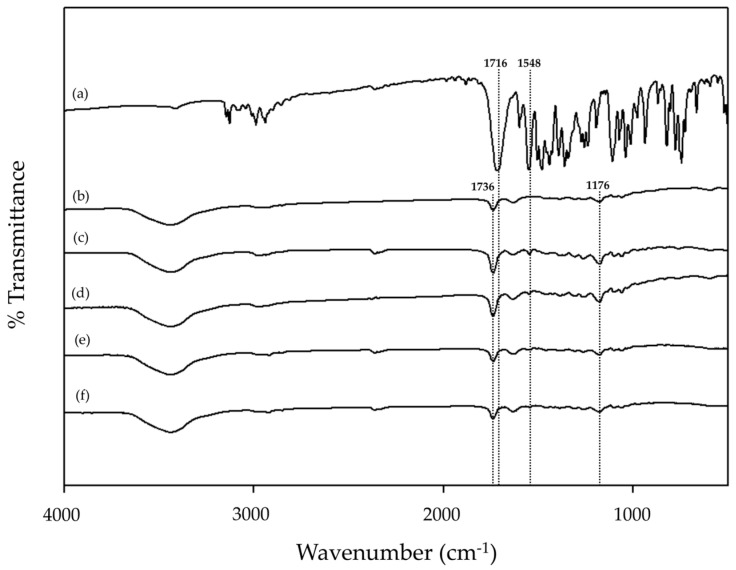
Fourier-transform infrared spectroscopic spectra of (a) PYR, (b) blank MPs, and PYR-MPs prepared at PHA:PYR ratio of (c) 5:1, (d) 10:1, (e) 15:1 and (f) 20:1.

Fourier-Transform infrared (FT-IR) spectroscopy was conducted to elucidate the chemical characteristics of PYR-MPs (Figure 3). The FT-IR spectrum of PYR exhibited a typical characteristic absorption peak at 1716.41 cm^−1^, corresponding to the C=O stretching vibration, along with distinct bands in the range of 1600–1470 cm^−1^, including a prominent peak at 1548.95 cm^−1^, attributed to the aromatic ring structure [34]. In the case of blank PHA MP, a strong absorption peak was observed at 1736.92 cm^−1^, corresponding to the ester carbonyl (C=O) stretching vibration, which is a representative characteristic peak of PHA [35]. Additionally, absorption bands in the range of 3000–2600 cm^−1^ were assigned to the stretching vibrations of CH_3_ and CH_2_ groups. The band at 2975.48 cm^−1^ is associated with CH_3_ stretching and presumably reflect interactions involving an adjacent carbonyl group [36]. Other peaks observed in the range of 1450–1000 cm^−1^ were attributed to CH_3_, CH_2_, and C–O groups [19]. For PYR-MPs, the characteristic peaks of PHA at 1736.92 cm^−1^ and 1176.58 cm^−1^ were still detectable, albeit with reduced intensity. The retention of the PHA backbone signals, together with the attenuated yet discernible the specific peak of PYR, indicates that PYR was successfully entrapped inside the PHA matrix without any significant chemical modifications.

### 3.4. Differential Scanning Calorimetric (DSC) Analysis

**Figure 4:**
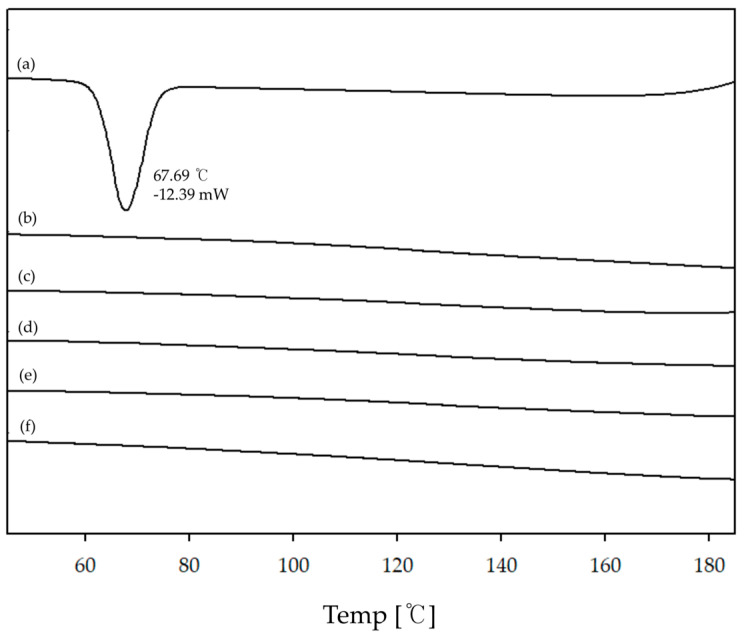
Differential scanning calorimetric thermograms of (a) PYR, (b) blank MPs, and PYR-MPs prepared at PHA:PYR ratios of (c) 5:1, (d) 10:1, (e) 15:1 and (f) 20:1.

Differential scanning calorimetry (DSC) was performed to investigate the thermal behavior of PYR and PYR-MPs (Figure 4). The DSC thermogram of free PYR exhibited a distinct endothermic melting peak at 67.69 °C. In contrast, this characteristic melting peak of PYR was absent in the thermogram of PYR-MPs over the corresponding temperature range. This absence may be associated with the amorphous or molecular dispersion of PYR within the MP matrices, possible physicochemical changes in PYR during microparticle preparation, its relatively low content in the MPs, and/or potential interactions between PYR and the PHA matrices.

### 3.5. X-Ray Diffraction (XRD) Analysis of PYR-MPs

**Figure 5:**
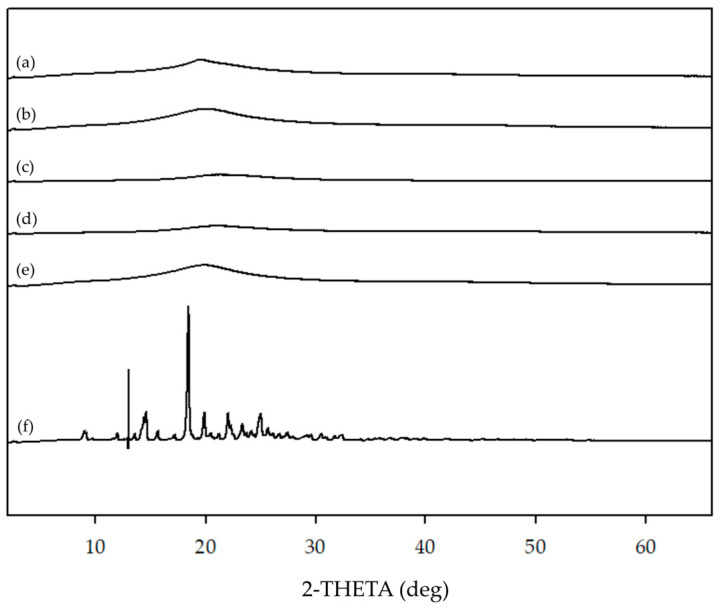
X-ray diffraction patterns of PYR-MPs prepared at PHA:PYR ratios of (a) 5:1, (b) 10:1, (c) 15:1, and (d) 20:1, along with (e) blank MPs and (f) PYR.

X-ray diffraction (XRD) analysis was conducted to evaluate the crystalline structures of PYR, blank MPs, and PYR-MPs (Figure 5). Free PYR exhibited a distinct diffraction peak at approximately 18.76°, whereas this characteristic peak was absent in all PYR-MPs. Instead, a broad halo pattern was observed around 20°, similar to that of the blank MPs, indicating the absence of well-defined crystalline peaks over the entire 2*θ* range [37,38]. Meanwhile, GC–MS analysis confirmed that the PHA consisted of 3HB and 4HB units, with a composition of approximately 75.4% and 24.6%, respectively (Figure S1). These GC–MS results indicate that the PHA used in this study possesses an amorphous state, which is presumably consistent with the amorphous characteristics observed from the XRD analysis.

### 3.6. Storage Stability of PYR-MPs

**Figure 6:**
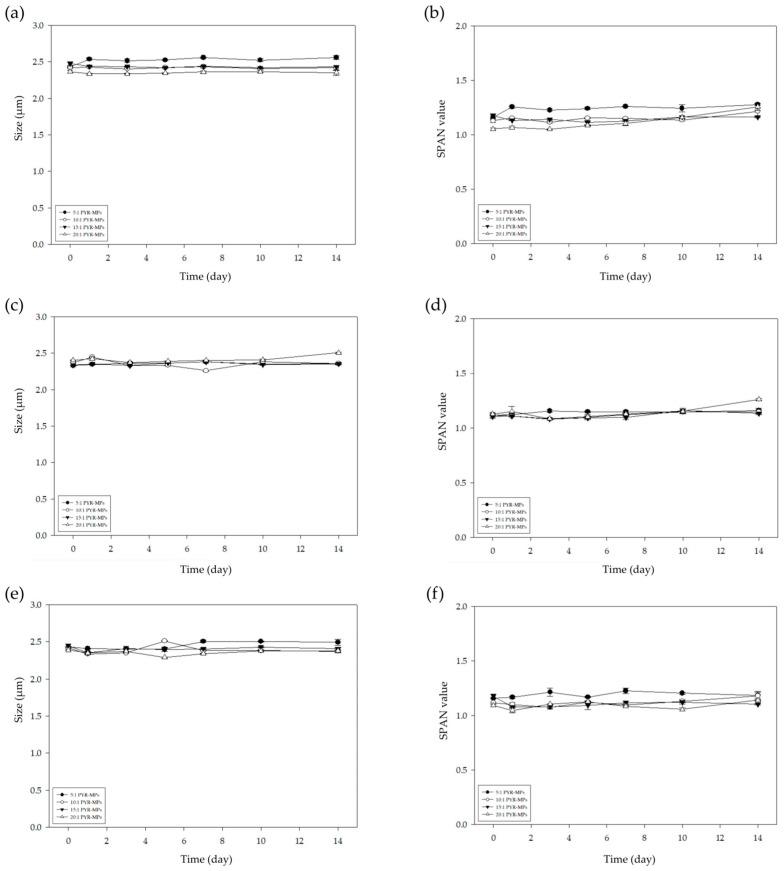
Storage stability of PYR-MPs at 4 °C, 25 °C and 54 °C over 14 days: (**a**) particle size and (**b**) SPAN value at 4 °C, (**c**) particle size and (**d**) SPAN value at 25 °C, (**e**) particle size and (**f**) SPAN value at 54 °C.

The storage stability of PYR-MPs was assessed by monitoring changes in particle size and size distribution over 14 days at 4, 25, and 54 °C (Figure 6). At 4 °C, no significant changes in particle size were observed, indicating excellent stability under the given condition. The magnitude of these variation was still minimal, remaining 5% of the initial particle size across all temperature conditions such as at 25 and at 54 °C. Furthermore, the SPAN values, reflecting particle size distribution, were consistently maintained within the narrow range of 1.1 to 1.2, with variation below 20%, indicating a stable and homogenous distribution. Taken together, the negligible extent of physical changes in PYR-MPs suggests that they exhibit robust colloidal stability across the tested storage conditions.

### 3.7. Photostability of PYR-MPs

**Figure 7:**
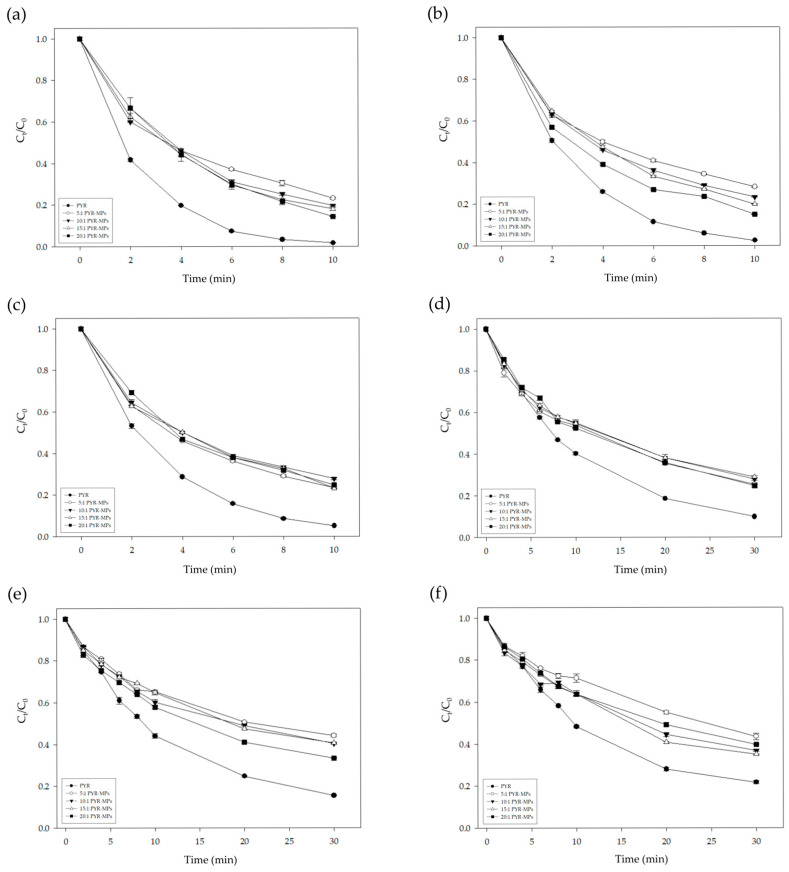
Photostability of free PYR and PYR-MPs at 50, 100, and 150 μg/mL under UV irradiation (36 W) at two irradiation distances: a short distance for (**a**–**c**) and long distance for (**d**–**f**), corresponding to the different concentrations of PYR: (**a**,**d**) 50 μg/mL, (**b**,**e**) 100 μg/mL, and (**c**,**f**) 150 μg/mL.

The photostability of PYR was evaluated at concentrations of 50, 100, and 150 μg/mL under UV irradiation (36 W) at various irradiation distances (Figure 7). Overall, all PYR-MPs exhibited markedly enhanced photostability compared to free PYR. At a short irradiation distance, the half-life (DT_50_) of free PYR ranged from 1.68 to 2.31 min depending on the concentration, whereas that of PYR-MPs increased to 3.61 to 6.50 min, representing more than a 2-fold extension. After 10 min of UV exposure, more than 94% of free PYR was degraded, indicating near-complete loss, whereas PYR-MPs reduced the degradation to 71.81 to 85.55% under the same conditions, demonstrating a clear photoprotective effect.

A similar behavior was observed at a long irradiation distance, indicating a relatively low energy of UV. The DT_50_ values of free PYR ranged from 8.89 to 13.25 min, whereas those of PYR-MPs increased to 15.49 to 29.64 min, indicating that the photodegradation-retarding effect of PYR-MPs was maintained regardless of the irradiation distance. After the UV exposure for 30 min, the degradation of free PYR reached 90.14%, 84.52%, and 78.26% at 50, 100, and 150 μg/mL, respectively. In comparison, PYR-MPs exhibited lower degradation levels of 71.19 to 75.30%, 55.88 to 66.61%, and 53.16 to 64.93% at the corresponding concentrations, further indicating improved photostability in given conditions. These findings suggest that encapsulation within the PHA matrix effectively protects PYR from UV-induced degradation, likely by providing a physical barrier that attenuates light penetration and limits direct exposure of the active ingredient. Although minor variations were observed among different formulation ratios, no consistent ratio-dependent trend was identified across the tested conditions, indicating that the photoprotective effect is largely independent of formulation composition within the studied range.

### 3.8. In Vitro Release of PYR from MPs

**Figure 8:**
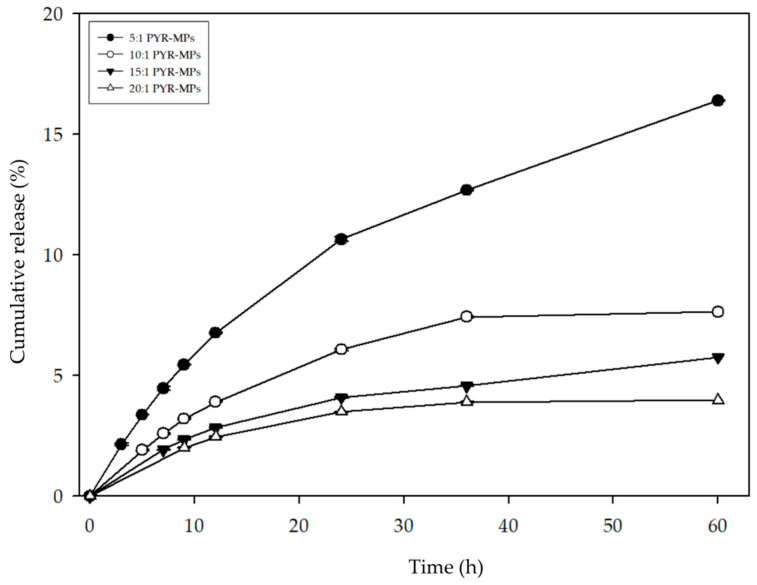
In vitro release profiles of PYR from MPs prepared at PHA:PYR ratios of 5:1, 10:1, 15:1 and 20:1.

In vitro release studies were conducted using a dialysis membrane method to investigate the time-dependent release profile of PYR form the PYR-MPs (Figure 8). As shown in the release profiles, all formulations exhibited a slow release pattern without any noticeable initial burst release, indicating the absence of significant surface absorbed drugs. Furthermore, the release rate was influenced by the PHA to PYR ratio. After 60 h, the cumulative release of PYR was relatively highest for the 5:1 formulation (16.38%), followed by the 10:1 formulation (7.63%), 15:1 formulation (5.75%), and 20:1 formulation (3.97%), which was similarly observed in the water-substituted medium (Figure S2 and Table S3). Overall, increasing the polymer amount resulted in a progressive decrease in drug release. Such release behavior of PYR can be attributed to the diffusion-controlled release, wherein lower polymer content facilitates drug diffusion through a less dense matrix, whereas higher PHA amount presumably leads to the formation of a more compact polymer network that restricts molecular mobility and retards drug release. This release-retarding phenomenon is consistent with our previous report [19,39], which demonstrated that the incorporation of PHA reduces PYR release rate in polymeric matrices. Taken together, these results indicated that the microparticulate system prolongs the release of PYR and that the release kinetics can be tuned by adjusting the formulation composition.

To elucidate the underlying release mechanism, various kinetic models were applied not only to the entire time range (0–60 h) but also to the initial (0–24 h) and later (24–60 h) stages (Table 3, Tables S1 and S2). During the initial stage (0–24 h), the Ritger–Peppas model generally provided the best fit for all formulation, with release exponent (*n*) values ranging from 0.555 to 0.773. These *n* values indicate non-Fickian transport, suggesting that both drug diffusion and polymer matrix relaxation contributed to the release process. In contrast, during the later stage (24–60 h), the Higuchi and Ritger–Peppas models better described the release behavior depending on the formulation, and the corresponding *n* values exhibited a decreasing pattern. Notably, increasing the polymer content led to lower *n* values, indicating a gradual shift toward Fickian diffusion. This transition suggests that the polymer matrix becomes more structurally stabilized, resulting in a release mechanism predominantly governed by diffusion rather than matrix relaxation.

Analysis of the entire time range (0–60 h, Table 3) further confirmed that the Higuchi and Ritger–Peppas models provided the best fit for all formulations with coefficients of determination (R^2^) exceeding 0.910, indicating that drug release is predominantly governed through the polymeric matrix. In addition, the release rate constant (*k*) decreased with increasing PHA content. For instance, in the Higuchi model, the *k* value decreased from 2.260 to 0.544 as the PHA:PYR ratio increased from 5:1 to 20:1. This trend quantitatively demonstrates that increased PHA content presumably leads to the formation of a denser and less permeable matrices, thereby retarding drug diffusion. Overall, the release mechanism of PYR-MPs exhibited a time-dependent transition from non-Fickian transport in the initial stage to predominantly diffusion-controlled behavior in the later stage. Notably, this diffusion-dominant behavior became more pronounced with increasing PHA content, highlighting the critical role of formulation composition and ratio in modulating release kinetics of PYR.

### 3.9. Antifungal Activity Assays of PYR

**Figure 9:**
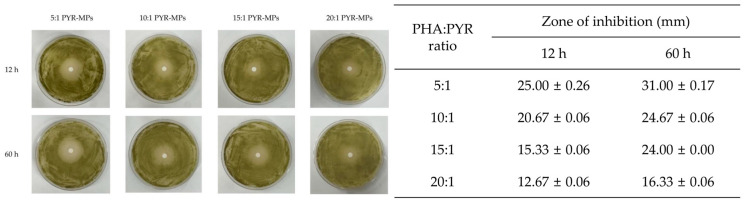
Antifungal activity of PYR-MPs prepared at PHA:PYR ratios of 5:1, 10:1, 15:1, and 20:1 against *Aspergillus oryzae,* evaluated by disk diffusion assay at 12 and 60 h.

The antifungal activity of the release samples collected from PYR-MPs at 12 and 60 h was evaluated using a disk diffusion assay (Figure 9). At 12 h, the inhibition zone was largest for the 5:1 PYR-MPs (25.00 ± 0.26 mm), followed by 10:1 (20.67 ± 0.06 mm), 15:1 (15.33 ± 0.06 mm), and 20:1 (12.67 ± 0.06 mm) PYR-MPs. A similar trend was observed at 60 h with the 5:1 PYR-MPs exhibiting the largest inhibition zone (31.00 ± 0.17 mm). The inhibition zones for the 10:1 and 15:1 PYR-MPs increased to 24.67 ± 0.06 mm and 24.00 ± 0.00 mm, respectively, while that of the 20:1 PYR-MPs also increased to 16.33 ± 0.06 mm, whereas no inhibition zone was observed for the control. Notably, all formulations exhibited larger inhibition zones at 60 h compared to those at 12 h. This enhancement in antifungal activity can be attributed to the increased amount of PYR released over time, as demonstrated in the release study, leading to higher local drug concentrations in the assay system. Taken together, these results confirm that the sustained release behavior of PYR-MPs effectively translates into prolonged and enhanced antifungal efficacy.

## 4. Discussion

Microbially derived PHAs are eco-friendly and biodegradable materials that offer a sustainable alternative to conventional synthetic polymers, particularly by mitigating environmental concerns associated with microplastic accumulation [40,41]. These attributes make PHAs highly attractive for agrochemical formulation, where environmental compatibility is increasingly critical. In this context, a PHA-based microparticulate system was developed for the encapsulation of the fungicide PYR, and its physicochemical properties, release behavior, and biological activity were systematically evaluated. Compared with delivery platforms based on conventional synthetic polymers, PHA-based microparticles may offer several advantages that are particularly relevant to agricultural applications. First, PHAs are biosynthesized by non-pathogenic microorganisms and are generally recognized as environmentally biodegradable polyesters. Accordingly, the use of PHA microparticles as carriers for fungicides or other pesticides may help mitigate concerns related to long-term environmental persistence, secondary microplastic formation, and polymer-associated ecological burden. This feature is especially important for field-applied pesticide formulations, including fungicides, because carrier materials are inevitably released into open environments. Second, our findings suggest that PHA-based microparticles can enhance photostability. Encapsulation within the PHA matrix may protect light-sensitive active ingredients from photodegradation, thereby preserving their chemical integrity and potentially prolonging their biological activity under irradiation. Together, these properties indicate that PHA-based microparticles represent a promising biodegradable delivery platform for pesticide formulations requiring both environmental compatibility and improved stability under field-relevant conditions.

In this study, the developed MPs enabled efficient encapsulation of PYR, and FT-IR and DSC analyses confirmed that the PYR was physically incorporated within the PHA matrix without undergoing chemical modification. In addition, photostability studies demonstrated that the PHA matrix acted as a protective barrier against UV irradiation, effectively suppressing photodegradation of PYR. From an analytical perspective, a validated HPLC method with high linearity and accuracy was employed to ensure reliable quantification of PYR throughout the study. The microparticles exhibited a relatively uniform size in the micrometer range with low SPAN values, indicating a narrow size distribution and good colloidal stability [42]. Such characteristics are advantageous for aqueous dispersion and are expected to facilitate practical application, including spray-based delivery in agricultural settings [43]. Comprehensive physicochemical characterization further suggested that PYR was predominantly encapsulated within the internal matrix rather than adsorbed on the particle surface. This is supported by the smooth surface morphology observed in SEM images and the absence of an initial burst release in the release profiles. Although this internal encapsulation resulted in a relatively slower release rate, antifungal activity assays against *Aspergillus oryzae* confirmed that sufficient bioactive concentrations were maintained to effectively inhibit fungal growth.

Taken together, the PYR-MPs exhibited structural stability, enhanced photostability, and controlled release behavior, all of which translated into sustained antifungal efficacy. These findings highlight the potential of this system as a versatile platform for the formulation of photolabile agrochemicals, including other pesticides, herbicides, and antifungal agents. Furthermore, the tunable release characteristics suggest broad applicability in agricultural and environmental applications.

## 5. Conclusions

Biodegradable PHA-based PYR MPs were developed with favorable formulation properties, enhanced photostability, and controlled release behavior. Encapsulation into MPs improved PYR photostability by approximately 1.5- to 3.0-fold compared with free PYR, depending on the PYR concentration and irradiation distance. PYR-MPs also exhibited a time-dependent shift from non-Fickian to diffusion-dominated release, with higher PHA contents slowing PYR diffusion through denser polymer matrices. Notably, the system retained the antifungal activity of PYR as a model fungicide. These findings highlight PHA-based MPs as biodegradable and eco-friendly delivery platforms for next-generation pesticide formulations.

## Figures and Tables

**Table 1:** Intra- and interday precision and accuracy of PYR measured by the developed HPLC method.

Concentration (μg/mL)	Precision (%)		Accuracy (%)	
	Intra-Day	Inter-Day	Intra-Day	Inter-Day
1.56	1.77	3.42	86.00	81.06
3.13	5.15	4.01	97.14	91.76
6.25	4.84	4.26	101.84	97.47
12.50	4.66	5.56	101.66	99.48
25.00	3.67	9.56	101.31	103.70
50.00	6.28	2.78	99.30	100.02
100.00	5.32	2.87	99.97	99.92
200.00	2.60	2.78	100.03	99.98

**Table 2:** Particle size, size distribution SPAN and encapsulation efficiency of PYR-MPs.

PHA:PYR Ratio	Size (µm)	SPAN Value	Encapsulation Efficiency (%)
Blank	2.40 ± 0.09	1.14 ± 0.05	-
5:1	2.41 ± 0.06	1.17 ± 0.04	74.13 ± 4.18
10:1	2.41 ± 0.04	1.12 ± 0.02	74.20 ± 2.14
15:1	2.41 ± 0.06	1.11 ± 0.03	75.45 ± 5.29
20:1	2.41 ± 0.07	1.11 ± 0.06	74.66 ± 1.52

**Table 3:** Release kinetic parameters of PYR from MPs over the entire period.

PHA:PYR Ratio	Zero-Order		First-Order		Higuchi		Ritger-Peppas		
	k	$R^{2}$	k	$R^{2}$	k	$R^{2}$	k	n	$R^{2}$
5:1	0.2624	0.9278	0.0029	0.9402	2.2596	0.9880	1.1551	0.6748	0.9855
10:1	0.1225	0.8179	0.0013	0.8241	1.1085	0.9550	0.8541	0.5793	0.9536
15:1	0.0846	0.859	0.0009	0.8655	0.7548	0.9915	0.7847	0.4968	0.9823
20:1	0.0579	0.6983	0.0006	0.7024	0.5442	0.9287	0.9543	0.3738	0.9102

## Data Availability

The original contributions presented in this study are included in the article/Supplementary Materials. Further inquiries can be directed to the corresponding authors.

## Supplementary Materials

The following supporting information can be downloaded at: https://www.mdpi.com/article/10.3390/polym18111380/s1, Figure S1: GC–MS chromatogram of PHA showing characteristic peaks corresponding to 3HB (6.77 min) and 4HB (7.81 min); Figure S2: In vitro release profiles of PYR from MPs prepared at PHA:PYR ratios of 5:1, 10:1, 15:1, and 20:1 in a water/ethanol (70:30, *v*/*v*) release medium; Table S1: Release kinetics of PYR from MPs over the initial period (0–24 h); Table S2: Release kinetics of PYR from MPs over the later period (24–60 h); Table S3: Release kinetics of PYR from MPs over the later period (0–60 h) in a water/ethanol (70:30, *v*/*v*) release medium.

## Abbreviations

The following abbreviations are used in this manuscript:

PHA	Polyhydroxyalkanoate
MPs	Microparticles
PYR	Pyraclostrobin
RSD	Relative standard deviation
LOQ	Limit of quantification
PVA	Polyvinyl alcohol
PSA	Particle size analyzer
SEM	Scanning electron microscope
FT-IR	Fourier-transform infrared spectroscopy
DSC	Differential scanning calorimeter
XRD	X-ray Diffraction
PBS	Phosphate-buffered saline
PDA	Potato dextrose agar
EE	Encapsulation efficiency

## References

[1] Lazarevic-Pasti T., Milankovic V., Tasic T., Petrovic S., Leskovac A. (2025). With or without you?—A critical review on pesticides in food. Foods.

[2] Kaur R., Singh D., Kumari A., Sharma G., Rajput S., Arora S., Kaur R. (2021). Pesticide residues degradation strategies in soil and water: A review. Int. J. Environ. Sci. Technol..

[3] Xie Z., Liang W., Xiong Q., Zhao Y., Cheng J., Li X., Zhao J. (2022). Acetalated dextran microparticles for the smart delivery of pyraclostrobin to control *Sclerotinia* diseases. Carbohydr. Polym..

[4] Qi Y., Zhang Z., Wang X., Long H., Pu L., Xu W. (2026). Temperature, pH and GSH triple-responsive pyraclostrobin nanopesticide based on Pluronic F127 and cinnamaldehyde-derived liposome to control tomato gray mold. Pestic. Biochem. Physiol..

[5] Shi X., Zhang Q., Deng W., Zhou R., Zhang J., Yu C., Wang T., Hu M., Yue X., Hao J. (2026). Cellulose-based nanocarrier for on-demand release and reduced aquatictoxicity of pyraclostrobin in fungal disease management. Chem. Eng. J..

[6] Xie H., Chen W., Liu Y., Wang T., Yan M., Zhao Y., Wang X. (2025). Graphene oxide/zein nanocomposites loaded with rotenone: Synergistically enhancing the pesticidal activity and biosafety of rotenone. Ind. Crops Prod..

[7] Zheng Z., Xu M., Cheng C., Du H., Peng F., Wang X., Yang Y., Zhang H., Hou W. (2025). Antifungal and controlled release properties of hymexazol-chitosan-graphene oxide composite. Int. J. Biol. Macromol..

[8] Chen L., Zhang W., Du H., Ding X., Li L., Chen H., Gao F., Cui B., Gao J., Cui H. (2024). Enhancing safety through the biodegradable pesticide microcapsules produced via melt emulsification and interfacial polymerization. Chem. Eng. J..

[9] Jiang F., Zhang J., Xu H., Lu Y., Wei S., Li Z. (2023). In situ preparation of monodisperse lignin-poly (lactic acid) microspheres for efficient encapsulation of 2,4-dichlorophenoxyacetic acid and controlled release. React. Funct. Polym..

[10] Yu B., Cheng J., Fang Y., Xie Z., Xiong Q., Zhang H., Shang W., Wurm F.R., Liang W., Wei F. (2024). Multi-stimuli-responsive, topology-regulated, and lignin-based nano/microcapsules from pickering emulsion templates for bidirectional delivery of pesticides. ACS Nano.

[11] Bhutkar S.P., Millard P.E., Urch H., Preece J.A., Zhang Z. (2025). Self-assembled microplastic-free microcapsules using aromatic bis-ureas with improved strength and tunable barrier properties for encapsulating cinmethylin. ACS Appl. Mater. Interfaces.

[12] Machado T.O., Grabow J., Sayer C., de Araujo P.H.H., Ehrenhard M.L., Wurm F.R. (2022). Biopolymer-based nanocarriers for sustained release of agrochemicals: A review on materials and social science perspectives for a sustainable future of agri- and horticulture. Adv. Colloid. Interface Sci..

[13] Lobel B.T., Baiocco D., Al-Sharabi M., Routh A.F., Zhang Z., Cayre O.J. (2024). Current challenges in microcapsule designs and microencapsulation processes: A review. ACS Appl. Mater. Interfaces.

[14] Peng X., Umer M., Pervez M.N., Hasan K.M.F., Habib M.A., Islam M.S., Lin L., Xiong X., Naddeo V., Cai Y. (2023). Biopolymers-based microencapsulation technology for sustainable textiles development: A short review. Case Stud. Chem. Environ. Eng..

[15] Cen J., Li L., Huang L., Jiang G. (2022). Construction of a photothermal controlled-release microcapsule pesticide delivery system. RSC Adv..

[16] Raut S.S., Sharma A., Mishra A. (2026). A decade of bibliometric and biotechnological advances in microbial polyhydroxyalkanoate (PHA) for biomedical applications: Developments in economical production, biosynthetic pathways, physicochemical properties, and therapeutic potential: A review. Int. J. Biol. Macromol..

[17] Jayalath S.U., de Alwis A.P. (2025). PHA, the greenest plastic so far: Advancing microbial synthesis, recovery, and sustainable applications for circularity. ACS Omega.

[18] Diniz M.S.D.F., Mourão M.M., Xavier L.P., Santos A.V. (2023). Recent biotechnological applications of polyhydroxyalkanoates (PHA) in the biomedical sector-a review. Polymers.

[19] Lee S.Y., Kim S.Y., Ku S.H., Park E.J., Jang D.J., Kim S.T., Kim S.B. (2022). Polyhydroxyalkanoate decelerates the release of paclitaxel from poly(lactic-co-glycolic acid) nanoparticles. Pharmaceutics.

[20] Heo J.Y., Sung M.K., Jang S., Kim H., Jeong Y., Jang D.J., Lee S.J., Kim S.B., Kim S.T. (2025). Surface functionalized polyhydroxyalkanoate nanoparticles via spytag-spycatcher system for targeted breast cancer treatment. Pharmaceutics.

[21] Song Y.K., Maeng J.E., Hwang H.R., Park J.S., Kim B.C., Kim J.K., Kim C.K. (2004). Determination of glimepiride in human plasma using semi-microbore high performance liquid chromatography with column-switching. J. Chromatogr. B.

[22] Yehia M., Saber E.A., Aly U.F., Naguib Y.W. (2025). Development and characterization of etoricoxib-loaded PLGA microparticles with tunable release profiles for the treatment of rheumatoid arthritis. J. Drug Deliv. Sci. Technol..

[23] Wang L., Liu W., Jiang Q., Wang X., Xu D., Fang Y., Wang S., Tang J. (2026). Span value as a critical quality attribute for PLGA microspheres: Controlling burst release and enhancing therapeutic efficacy via wet sieving. Pharmaceutics.

[24] Sachan R.S.K., Kumar A., Karnwal A., Paramasivam P., Agrawal A., Ayanie A.G. (2025). Screening and characterization of PHA producing bacteria from sewage water identifying *Bacillus* paranthracis RSKS-3 for bioplastic production. BMC Microbiol..

[25] Guo Q., Liu Y., Huang Y., Hu G., Tang G., Zhang X., Yan W., Xiao J., Yan G., Shi J. (2025). Nanocapsules bearing imide polymer as wall material for pH-responsive and synergistic fungicidal activity. Chem. Eng. J..

[26] Zhang N., Xiao Y., Hu S., Chen Q., Huang Y., Li M., Jin Z., Chen H., Wu W., Wang J. (2024). Improving UV protection and retention of photosensitive agrochemicals: Innovative polyurethane-CeO2 hybrid pesticide microcapsules. Chem. Eng. J..

[27] Xiao R., Cao Z., Yuan B., Chen Y., Li X., Dong J., Du X. (2025). Continuous and scalable preparation of environmentally friendly controlled-release pesticide nanocapsules using low-energy-input microfluidics. Chem. Eng. J..

[28] Wang A., Cheng X., Ding C., Zhao P., Cao C., Cao L., Yu M., Huang Q. (2026). Pectin-modified pyraclostrobin nanocapsules: Targeted delivery of pesticides for improved control of stem base rot of wheat. Int. J. Biol. Macromol..

[29] Liu J., Wang X., Chang J., Du P., Wu J., Hou R., Zhu S., Liu P., Miao X., Zhang P. (2024). Green synthesized lignin nanoparticles for the sustainable delivery of pyraclostrobin to control strawberry diseases caused by Botrytis cinerea. Int. J. Biol. Macromol..

[30] Liang Y., Song J., Dong H., Huo Z., Gao Y., Zhou Z., Tian Y., Li Y., Cao Y. (2021). Fabrication of pH-responsive nanoparticles for high efficiency pyraclostrobin delivery and reducing environmental impact. Sci. Total Environ..

[31] Hu F., Tu X.F., Thakur K., Hu F., Li X.L., Zhang Y.S., Zhang J.G., Wei Z.J. (2019). Comparison of antifungal activity of essential oils from different plants against three fungi. Food Chem. Toxicol..

[32] Alshannaq A., Henning M., Dixon J., Riley C., Choi D., Yu J.H., Safdar N. (2025). Potent antimicrobial activity of *Aspergillus oryzae* fermentate against toxigenic strains of Clostridioides difficile. Antibiotics.

[33] Charbe N., Baldelli S., Cozzi V., Castoldi S., Cattaneo D., Clementi E. (2016). Development of an HPLC-UV assay method for the simultaneous quantification of nine antiretroviral agents in the plasma of HIV-infected patients. J. Pharm. Anal..

[34] Yin M.-M., Zheng Y., Chen F.-L. (2018). Pyraclostrobin-loaded poly (lactic-co-glycolic acid) nanospheres: Preparation and characteristics. J. Integr. Agric..

[35] El-Malek F.A., Farag A., Omar S., Khairy H. (2020). Polyhydroxyalkanoates (PHA) from *Halomonas pacifica* ASL10 and *Halomonas salifodiane* ASL11 isolated from mariout salt lakes. Int. J. Biol. Macromol..

[36] Sathiyanarayanan G., Bhatia S.K., Song H.S., Jeon J.M., Kim J., Lee Y.K., Kim Y.G., Yang Y.H. (2017). Production and characterization of medium-chain-length polyhydroxyalkanoate copolymer from Arctic psychrotrophic bacterium Pseudomonas sp. PAMC 28620. Int. J. Biol. Macromol..

[37] Majka T.M., Raftopoulos K.N., Hebda E., Szeligowski A., Zastawny O., Guzik M., Pielichowski K. (2024). PHB+aPHA blends: From polymer bacterial synthesis through blend preparation to final processing by extrusion for sustainable materials design. Materials.

[38] Xiong H., Liu X., Xu J., Zhang X., Luan S., Huang Q. (2020). Fungicidal effect of pyraclostrobin against *Botrytis cinerea* in relation to its crystal structure. J. Agric. Food Chem..

[39] Murueva A.V., Shershneva A.M., Shishatskaya E.I., Volova T.G. (2023). Characteristics of microparticles based on resorbable polyhydroxyalkanoates loaded with antibacterial and cytostatic drugs. Int. J. Mol. Sci..

[40] Behera S., Priyadarshanee M., Das S. (2022). Polyhydroxyalkanoates, the bioplastics of microbial origin: Properties, biochemical synthesis, and their applications. Chemosphere.

[41] Hadri S.H., Tareen N., Hassan A., Naseer M., Ali K., Javed H. (2025). Alternatives to conventional plastics: Polyhydroxyalkanoates (PHA) from microbial sources and recent approaches—A review. Process Saf. Environ. Prot..

[42] Santos J., Trujillo-Cayado L.A., Carrillo F., Lopez-Castejon M.L., Alfaro-Rodriguez M.C. (2022). Relation between droplet size distributions and physical stability for zein microfluidized emulsions. Polymers.

[43] Chan D.H.H., Kynaston E.L., Lindsay C., Taylor P., Armes S.P. (2021). Block copolymer nanoparticles are effective dispersants for micrometer-sized organic crystalline particles. ACS Appl. Mater. Interfaces.

