# Supplementary material for: Polyhydroxyalkanoate-Based Microparticles for Enhanced Photostability and Controlled Release of Pyraclostrobin
Source: Polymers (Basel). 2026 Jun 2;18(11):1380. doi: 10.3390/polym18111380 (PMC13259420; doi:10.3390/polym18111380)
Supplement: Supplementary file 1 [file polymers-18-01380-s001.zip › Table S1, S2, S3.pdf]

Table S1. Release kinetics of PYR from MPs over the initial period (0–24 h).

PHA:PYR ratio	Zero-Order		First-Order		Higuchi		Ritger-Peppas		
	k	R^2	k	R^2	k	R^2	k	n	R^2
5:1	0.4295	0.9656	0.0046	0.9723	2.2164	0.9623	0.9621	0.7729	0.9945
10:1	0.2434	0.9568	0.0025	0.9613	1.2396	0.9689	0.6145	0.7318	0.9914
15:1	0.163	0.9194	0.0017	0.9233	0.9108	0.9764	0.6145	0.6027	0.9897
20:1	0.1417	0.9242	0.0014	0.9270	0.7090	0.9981	0.6015	0.5551	0.9943

Table S2. Release kinetics of PYR from MPs over the later period (24–60 h).

PHA:PYR ratio	Zero-Order		First-Order		Higuchi		Ritger-Peppas		
	k	R^2	k	R^2	k	R^2	k	n	R^2
5:1	0.1589	0.9994	0.0018	0.9998	2.0280	0.9987	2.3572	0.4725	0.9982
10:1	0.0385	0.6931	0.0385	0.694	0.5109	0.7470	2.9168	0.2421	0.7901
15:1	0.0466	0.9973	0.0466	0.9971	0.5923	0.9876	1.2232	0.3753	0.9818
20:1	0.0120	0.7232	0.0001	0.7235	0.1589	0.7752	2.2940	0.1377	0.8192

Table S3. Release kinetics of PYR from MPs over the later period (0–60 h) in a water/ethanol (70:30, v/v) release medium.

PHA:PYR ratio	Zero-Order		First-Order		Higuchi		Ritger-Peppas		
	k	R^2	k	R^2	k	R^2	k	n	R^2
5:1	0.2289	0.8333	0.0025	0.8422	2.0341	0.9449	0.9815	0.7040	0.9530
10:1	0.1047	0.8390	0.0011	0.8455	0.9437	0.9724	0.7761	0.5623	0.9591
15:1	0.0826	0.8515	0.0009	0.8569	0.7368	0.9818	0.7318	0.5061	0.9722
20:1	0.0407	0.7320	0.0004	0.7359	0.3764	0.9415	0.7406	0.3411	0.8875
