# Supplementary figures and images for: Polyhydroxyalkanoate-Based Microparticles for Enhanced Photostability and Controlled Release of Pyraclostrobin
Source: Polymers (Basel). 2026 Jun 2;18(11):1380. doi: 10.3390/polym18111380 (PMC13259420; doi:10.3390/polym18111380)

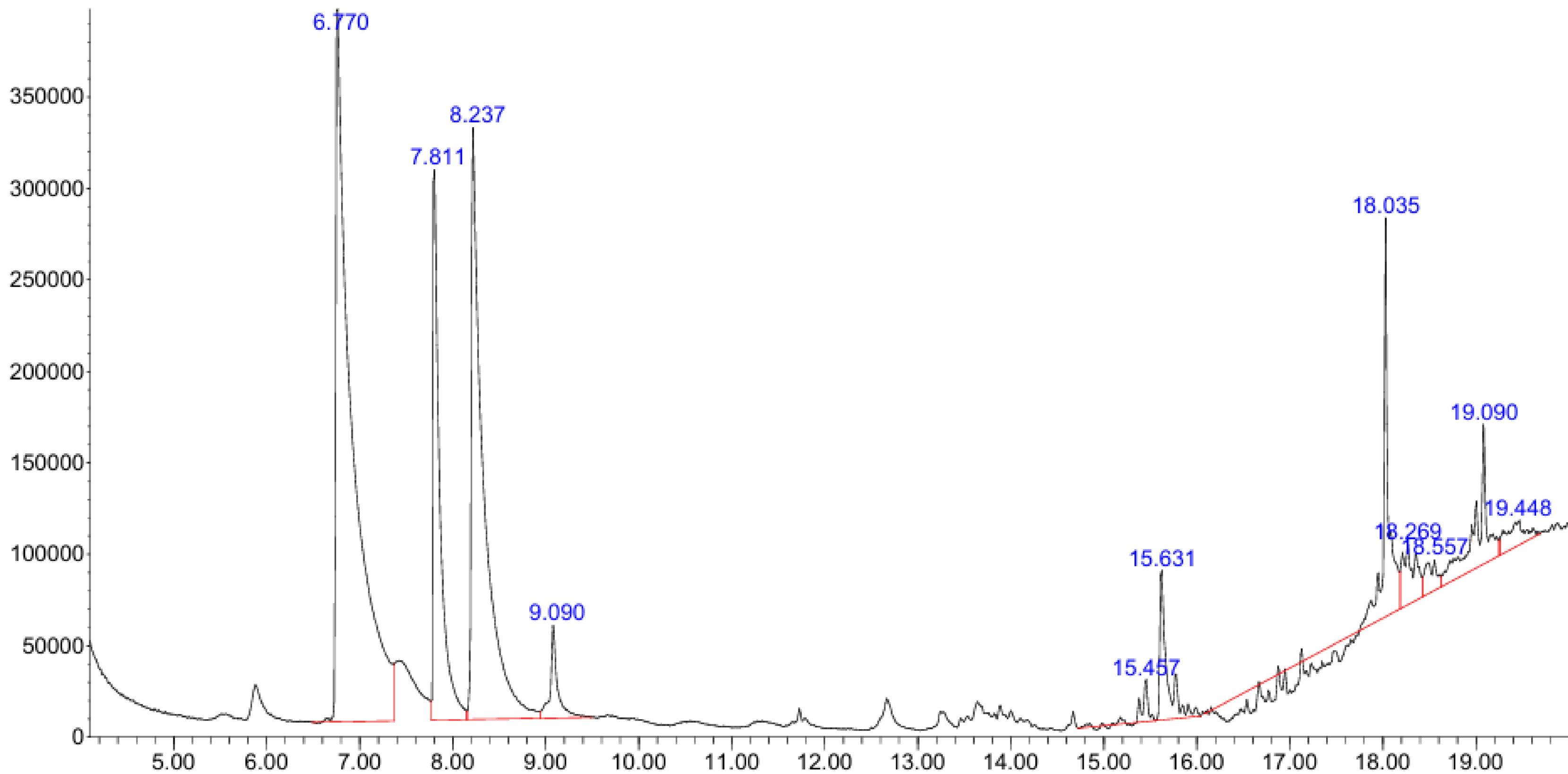

Supplement: Supplementary file 1 [file polymers-18-01380-s001.zip › Figure S1.pdf]

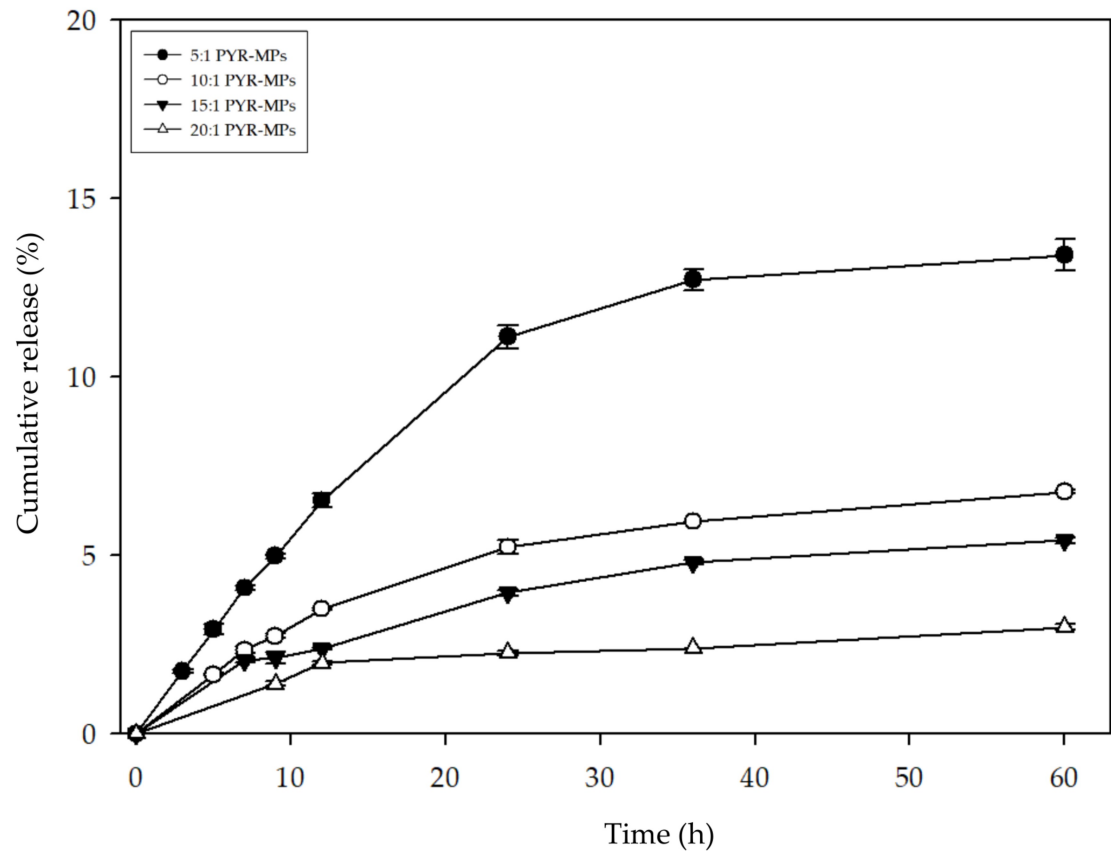

Supplement: Supplementary file 1 [file polymers-18-01380-s001.zip › Figure S2.pdf]
